# Predicting Australian Adults at High Risk of Cardiovascular Disease Mortality Using Standard Risk Factors and Machine Learning

**DOI:** 10.3390/ijerph18063187

**Published:** 2021-03-19

**Authors:** Shelda Sajeev, Stephanie Champion, Alline Beleigoli, Derek Chew, Richard L. Reed, Dianna J. Magliano, Jonathan E. Shaw, Roger L. Milne, Sarah Appleton, Tiffany K. Gill, Anthony Maeder

**Affiliations:** 1 Flinders Digital Health Research Centre, College of Nursing & Health Sciences, Flinders University, Adelaide SA 5001, Australia; stephanie.champion@flinders.edu.au (S.C.); alline.beleigoli@flinders.edu.au (A.B.); anthony.maeder@flinders.edu.au (A.M.); 2 Chifley Business School, Torrens University, Australia, Adelaide, SA 5000, Australia; 3 Caring Futures Institute, Flinders University, Adelaide, SA 5001, Australia; 4 College of Medicine and Public Health, Flinders University, Adelaide, SA 5001, Australia; derek.chew@flinders.edu.au (D.C.); richard.reed@flinders.edu.au (R.L.R.); 5 Baker Heart and Diabetes Institute, Melbourne, VIC 3004, Australia; Dianna.Magliano@baker.edu.au (D.J.M.); Jonathan.Shaw@baker.edu.au (J.E.S.); 6 School of Public Health and Preventive Medicine, Monash University, Melbourne, VIC 3004, Australia; 7 School of Life Sciences, La Trobe University, Melbourne, VIC 3086, Australia; 8 Cancer Epidemiology Division, Cancer Council Victoria, 615 St Kilda Road, Melbourne, VIC 3004, Australia; Roger.Milne@cancervic.org.au; 9 Centre for Epidemiology and Biostatistics, Melbourne School of Population and Global Health, The University of Melbourne, 207 Bouverie Street, Melbourne, VIC 3010, Australia; 10 Precision Medicine, School of Clinical Sciences at Monash Health, Monash University, Clayton, VIC 3168, Australia; 11 Flinders Health and Medical Research Institute (Sleep Health)/Adelaide Institute for Sleep Health (AISH), College of Medicine and Public Health, Flinders University, Adelaide, SA 5042, Australia; sarah.appleton@flinders.edu.au; 12 Adelaide Medical School, The University of Adelaide, Adelaide, SA 5005, Australia; tiffany.gill@adelaide.edu.au

**Keywords:** artificial intelligence, machine learning, clinical decision support, cardiovascular disease, cardiovascular risk factors, risk prediction

## Abstract

Effective cardiovascular disease (CVD) prevention relies on timely identification and intervention for individuals at risk. Conventional formula-based techniques have been demonstrated to over- or under-predict the risk of CVD in the Australian population. This study assessed the ability of machine learning models to predict CVD mortality risk in the Australian population and compare performance with the well-established Framingham model. Data is drawn from three Australian cohort studies: the North West Adelaide Health Study (NWAHS), the Australian Diabetes, Obesity, and Lifestyle study, and the Melbourne Collaborative Cohort Study (MCCS). Four machine learning models for predicting 15-year CVD mortality risk were developed and compared to the 2008 Framingham model. Machine learning models performed significantly better compared to the Framingham model when applied to the three Australian cohorts. Machine learning based models improved prediction by 2.7% to 5.2% across three Australian cohorts. In an aggregated cohort, machine learning models improved prediction by up to 5.1% (area-under-curve (AUC) 0.852, 95% CI 0.837–0.867). Net reclassification improvement (NRI) was up to 26% with machine learning models. Machine learning based models also showed improved performance when stratified by sex and diabetes status. Results suggest a potential for improving CVD risk prediction in the Australian population using machine learning models.

## 1. Introduction

Cardiovascular disease (CVD) is the leading cause of death in Australia [1]. Many cardiovascular disease risk factors are modifiable and, with early diagnosis and intervention of individuals at higher risk, CVD mortality and morbidities are largely preventable [2]. Risk prediction models that combine known CVD predictors, such as hypertension, cholesterol, age, smoking, and diabetes, have traditionally been used to identify those at greatest risk. The Framingham, Systematic COronary Risk Evaluation (SCORE), and QRISK models are commonly used in the UK, US, Australia, and New Zealand to inform public policy and clinical guidelines [3,4]. 

Two of the most pertinent limitations of established risk prediction models are: (1) traditional predictive models based on personal health information use simple regression fitting approaches that cannot assume nonlinear relationships between the predictors and outcome measures, which oversimplifies the associations between CVD risk factors and outcomes, thus reducing the accuracy of predictions [5], and (2) there is a limited generalizability of models to accurately predict the risk of CVD in diverse populations and across countries [3,4]. For example, the Framingham Risk Score, one of the most commonly used and widely validated models worldwide, is derived from a largely Caucasian population of European descent, and may be less accurate for some high-risk groups, such as individuals with diabetes, socio-economically disadvantaged populations [6], and Australian females [7]. 

Machine learning (ML) is a widely accepted computational technique that can address the nonlinear relationships between the risk factors and outcome measures [8]. It also presents an opportunity to improve the robustness and generalizability of prediction models for CVD by constructing phenotypical cohort-based risk models [9]. The potential of improved accuracy in predicting CVD risk using machine learning approaches, compared to the Framingham Risk Score, has been investigated in several international cohorts [5,10,11,12]. Using large UK cohorts, Weng et al. [5] utilized four machine learning models (logistic regression, random forest, gradient boosting machines, neural networks) to predict CVD events, and Alaa et al. [10] tested the potential of an automated machine learning framework (AutoPrognosis) for predicting CVD events. In the US, Ambale-Venkatesh et al. [11] and Kakadiaris et al. [12] also used random forest and support vector machine, respectively, to predict CVD events and mortality in US populations. A 2020 meta-analysis assessing the predictive ability of machine learning algorithms for cardiovascular diseases found promising potential in ML approaches [13]. The Framingham Risk Score is recommended for use in Australia to predict CVD risk but has been found to have limited accuracy for some Australian sub-populations [7,14]. A recent Australian study based on 5453 participants showed that the widely accepted 2008 Framingham model has overestimated the CVD risk by 29.7% in men and 7.2% in women [14]. 

This investigation aims to improve CVD risk prediction for the Australian popula-tion by applying different ML techniques to the risk factors used by the 2008 Framingham Risk Score. To our knowledge, these ML based CVD risk prediction models have not previously been applied to Australian population cohorts.

ML is mainly classified into two categories: supervised and unsupervised. If a set of training data is available and the classifier is designed based on that prior information, then it is known as supervised learning, whilst in unsupervised learning no prior training information is available. [15]. The performance of four supervised ML techniques used to derive risk prediction models for cardiovascular deaths for three Australian sub-populations were compared, individually and in combination, in male and female sub-cohorts, and in a diabetes cohort. This study is an applied public health epidemiological research approach using tools of computational modelling (machine learning models). It will be a novel contribution to public health.

## 2. Materials and Methods

### 2.1. Study Sample

Data from the North West Adelaide Health Study (NWAHS) [16], the Australian Diabetes, Obesity, and Lifestyle (AusDiab) study [17], and the Melbourne Collaborative Cohort Study (MCCS) [18] were used in this analysis. Detailed descriptions of the NWAHS [16], AusDiab [17], and the MCCS [18] cohorts, recruitment, response rates, and data collection procedures have been previously published. 

### 2.2. Risk Factors and CVD Mortality

Eight core baseline variables (age, sex, total cholesterol, high-density lipoprotein (HDL) cholesterol, systolic blood pressure, hypertension medication, diabetes, and smoking status, Table 1, were used to derive all the CVD risk prediction models. The outcome measure used was CVD mortality. Non-fatal CVD events were excluded from the outcome measure as that information was not available in all three datasets. CVD mortality was defined as deaths that occurred within 15 years of baseline, with CVD listed as the primary or secondary cause of death based on International Classification of Diseases (ICD) from the 9th (390–459) and 10th (I00–I99) revisions. 

### 2.3. Participant Numbers and Missing Values

The study population characteristics are reported in Table 2. Out of 4056 NWAHS participants, we excluded 326 people with a previous CVD history, 6 with missing CVD history data, and 70 with missing CVD outcome data. This led to a sample of 3654 participants. For the AusDiab study, out of 11,247 participants, 938 with a previous CVD history, 142 with missing CVD history data, and 17 with missing CVD outcome data were excluded, leaving 10,150 participants for the analysis. Of the 41,513 MCCS participants, 7035 participants with a previous CVD history and 1867 with missing CVD outcome were excluded. This resulted in 32,611 participants for the analysis.

The missing values in the risk factor variables were imputed using the missRanger algorithm [19]. The missRanger algorithm uses random forest trained imputations on observed data to predict continuous and categorical missing values. Random forest-based imputations perform better than the traditional imputation methods for epidemiologic datasets with missing data [20]. Imputation models that treat continuous variables as linear may be less able to account for complex interactions and non-linear relationships between the variables, compared to random forest-based imputations.

### 2.4. Framingham Risk Prediction Model

For the Framingham model, the CVD risk score was calculated using the eight baseline variables (mentioned previously) included in the 2008 Framingham model [21]. The Framingham model assigns a person to the low-risk group if the score is < 20 and to the high-risk group if the score is ≥ 20. As the Framingham equation was designed to estimate 10-year CVD risk and in this study the follow up data is for 15 years, we have linearly transformed the 10-year risk of the Framingham model into 15-year risk [13]. Thus, the Framingham score risk threshold became 30 instead of 20.

### 2.5. Machine Learning Risk Prediction Model

**Figure 1:**
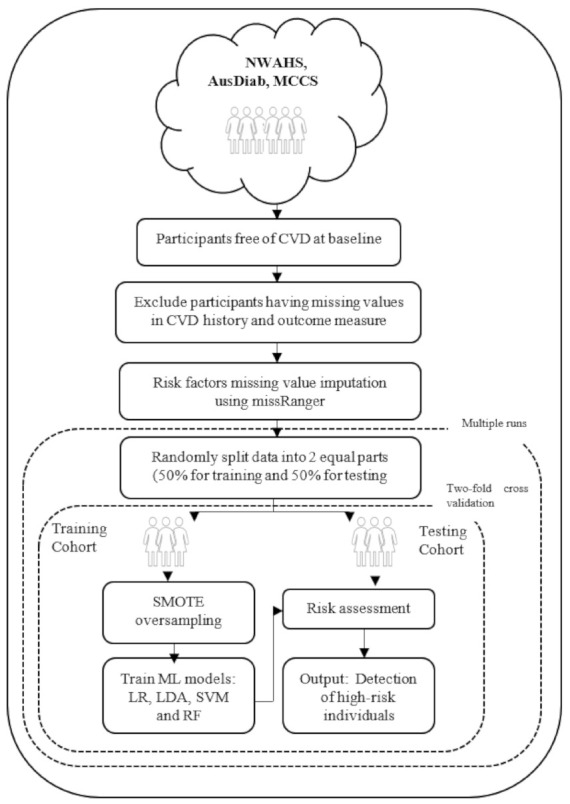
Flowchart describing the machine learning approach. CVD indicates cardiovascular disease; Synthetic Minority Over Sampling Technique (SMOTE); Machine Learning (ML); logistic regression (LR); linear discriminant analysis (LDA); support vector machine with linear kernel (SVM); random forest (RF).

Figure 1 shows an overview of the machine learning approach used. The algorithm starts with input of the cohort data (NWAHS, AusDiab, or MCCS). Input variables (eight baseline variables mentioned previously) were normalized to zero mean and unit variance within each dataset to ensure each variable had the same influence on the cost function in designing the machine learning models. This was done separately on training and testing data. 

The three cohort datasets were severely imbalanced. The number of participants who had died due to CVD on or before 15 years follow-up (minority class) was much smaller than the number of participants alive at 15 years follow-up (majority class). The minority class percentage were 3.3, 3.4, and 1.6 for the NWAHS, AusDiab, and the MCCS, respectively (shown in Table 1). As this imbalance affects the decision boundary of the machine learning models and results in poor performance, the Synthetic Minority Over Sampling Technique (SMOTE) algorithm was used [22] to oversample the minority class and balance the data in the training set. 

Four popular machine learning models were applied to each cohort: logistic regression (LR), linear discriminant analysis (LDA), support vector machine with linear kernel (SVM), and random forest (RF) [15,23]. The performance of each model was measured using the testing data. To maximize the models’ robustness and generalizability, two-fold cross validation was used. For this approach, the original data was randomly split into two equal sized subsets: a training set to train the models, and a testing set to evaluate them. Then the sets were swapped and the process was repeated. The two results were averaged. To ensure stable classification results, the overall process was repeated 10 times for each of the four models and the results were averaged. In addition, to test the generalizability of the machine learning models, another experiment was conducted by taking AusDiab and the MCCS as the training set and the NWAHS as the external validation set. 

### 2.6. Software

The programming for the Framingham score calculation and preprocessing of the data (participants exclusion process) was completed in MATLAB R2018b [24]. Missing value imputation was done in the R 3.6.1 using the Ranger package (R Foundation for Statistical Computing, Vienna, Austria). Standardization of features and machine learning algorithms were implemented using the Scikit-learn library in Python (Python Software Foundation, Wilmington, United States) [25].

### 2.7. Statistical Analysis 

The performance of the Framingham model was evaluated using area-under-curve (AUC) score, sensitivity (Sen), specificity and precision based on the prediction equation, and the risk threshold described previously. Then, performance of the machine learning models was analyzed, compared with those of Framingham, and the categorial net reclassification improvement (NRI) for the paired models was calculated. The optimal threshold for classification was found from receiver operating characteristic (ROC) Curve. The optimal threshold was the point where there was the maximum difference between sensitivity and specificity. Variable importance was assessed using a random forest technique to rank the features according to their contributions to the predictions. The random forest variable ranking method has been successfully used for similar studies [12,26]. The dependent variable for the models was CVD mortality.

### 2.8. Ethics Approval

The NWAHS was approved by the Human Research Committee of the Queen Elizabeth Hospital in South Australia, the AusDiab study was approved by the Alfred Human Research Ethics Committee, and the MCCS was approved by the Cancer Council Victoria’s Human Research Ethics Committee. 

## 3. Results

The prediction accuracy of all models, for the individual and combined cohorts, according to the AUC performance measure, is shown in Table 3. For the NWAHS and AusDiab cohorts, all four of the ML models achieved significantly better performance than the Framingham model for predicting CVD deaths. For the MCCS, except for the Logistic Regression model, all other ML models achieved slightly better performance than the Framingham model. When all three study populations were combined (46,315 participants, 982 CVD deaths) the Logistic Regression and Linear Discriminant Analysis models performed significantly better than the Framingham model for predicting CVD deaths.

The classification analysis outcomes can be found in Table 4. For the NWAHS, the Framingham model predicted 50 out of 121 CVD deaths correctly (Sen 41.3%, PPV 14.0%), compared to 98 deaths using the Support Vector Machine model (Sen 80.7%, PPV 13.0%). For AusDiab, the Framingham model correctly predicted 195 out of 341 deaths (Sen 57.2%, PPV 14.4%) compared to 291 with the ML model, Linear Discriminant Analysis (Sen 85.2%, PPV 15.7%). For the MCCS, the Framingham model correctly predicted 162 out of 520 deaths (Sen 31.2%, PPV 5.6%) compared to 425 with the ML model, Random Forest (Sen 81.6%, PPV 3.5%). Even in the combined cohort, the Framingham model correctly predicted fewer CVD deaths (408 out of 982 deaths, Sen 41.5%, PPV 8.8%) compared to all four ML models. The Logistic Regression model performed best (796 out of 982 deaths, Sen 81.0%, PPV 8.1%). The categorical NRI between the Framingham model and each of the machine learning models are also shown in Table 4. For the machine learning models, an NRI up to 29%, 24%, and 22% for the NWAHS, AusDiab, and the MCCS, respectively, were achieved when compared with the Framingham model. For the aggregated cohort, the machine models achieved an NRI up to 26%. 

A random forest technique [26] was used to predict variable importance. Table 5 lists the variables ranked according to their contribution to prediction for the individual datasets and the combined cohort. For all three individual datasets and in the combined dataset, age appeared to be the most important predictor that was linked to a higher CVD risk, followed by systolic blood pressure.

### 3.1. Sex Stratification

An analysis of the prediction accuracy of all models when applied to the combined cohort stratified by sex found that machine learning models returned higher AUC scores when compared to the Framingham model for male and female populations (Table 6). The classification performance of the Framingham model was less in females compared to males, correctly predicting 75 out of 481 CVD deaths for females (Sen 15.6, PPV 15.7), compared to 333 deaths out of 501 deaths for males (Sen 66.3, PPV 8.0). The ML models performed significantly better at predicting CVD male and female deaths than the Framingham model. In the male cohort, the Linear Discriminant Analysis and Support Vector Machine models were able to predict 382 out of 501 CVD deaths while, in the female cohort, Logistic Regression and Support Vector Machine models correctly predicted 402 out of 481 CVD deaths. NRI were up to 6.5% and 48.7% for the male and female cohorts, respectively, compared to the Framingham model. Details of this classification analysis can be found in Table 6 and Table 7.

### 3.2. Diabetes Stratification

Among 46,315 participants in the combined cohort, a total of 3791 participants reported a diagnosis of diabetes at baseline. All machine learning models achieved significant improvement in prediction accuracy compared to the Framingham model for the diabetes cohort and non-diabetes cohort (Table 8). Additionally, the four ML models performed significantly better in classification performance than the Framingham model for the diabetes cohort and non-diabetes cohort (Table 9). The Framingham model correctly predicted only 163 CVD deaths out of 231 deaths in the diabetes cohort (Sen 70.1%, PPV 11.1) and 245 CVD deaths out of 751 in the non-diabetes cohort (Sen 32.6%, PPV 7.7). In comparison, the Linear Discriminant Analysis model performed best in both diabetes and non-diabetes cohorts, correctly predicting 185 out of 231 CVD deaths (Sen 80.0%, PPV 16.1) in the diabetes cohort and 629 out of 751 CVD deaths (Sen 84.0%, PPV 5.6) in the non-diabetes cohort. For the ML models, NRI were up to 18.7% and 31.2% for the diabetes and non-diabetes cohorts, respectively, compared to the Framingham model. 

### 3.3. External Validation

To evaluate the performance of ML models on unseen data, prediction models were developed using a combined AusDiab and MCCS dataset as the training set and the NWAHS as an the external validation set. The comparison of AUC score, classification results (sensitivity, specificity and precision), and NRI are shown in Table 10. All four machine learning models achieved significant improvement in performance (AUC score, sensitivity, precision) compared to the Framingham model when the model was trained using combined AusDiab and MCCS data and tested on NWAHS data. The support vector machine achieved an AUC score of 0.880 and sensitivity of 72.5, much higher than the Framingham model (AUC 0.837, Sen 41.3). The highest NRI was achieved by the linear discriminant analysis model (29.4). Even when data was stratified based on sex and diabetic diagnosis, the machine learning models performed better than Framingham model. 

## 4. Discussion

This study evaluated the potential of four machine learning CVD risk prediction models for predicting CVD mortality risk in Australian population cohorts, compared with the Framingham model, using eight traditional risk factors. We have validated the ML models both internally (two fold cross validation) and externally (training on combined AusDiab and MCCS data and tested on unseen NWAHS data). To our knowledge, this is the first multiple dataset and multiple sub-cohort study applying machine learning to the Australian population, demonstrating improved performance of predicting CVD risk with machine learning models.

All four machine learning models performed significantly better than the Framingham model at identifying individuals at very high risk of CVD in the Australian population in terms of discrimination, risk classification, and decision curve analysis. Machine learning models improved prediction (AUC score) by up to 5.1% in the aggregated cohort (NWAHS, AusDiab, and MCCS combined cohort), 1.9% in the male cohort, 3.5% in the female cohort, 9.1% in the diabetes cohort, and 5.5% in the non-diabetes cohort (See Table 3, Table 6 and Table 8). 

Additionally, this study found that machine learning models detected up to 68% more ‘true positive’ female cases than the Framingham model and identified 49% net reclassification improvement with the ML models (See Table 7). Recent investigations have shown disparities in the care received by Australian women with CVD compared to Australian men [27]. This can in part be attributed to underdiagnosis or delay in diagnosis of women, resulting from sex differences in CVD pathophysiological mechanisms, clinical presentation, and course of disease [27], and a higher prevalence of comorbid conditions in female CVD patients [28]. Framingham models have been found to underestimate CVD risk for women [27]. Machine learning models to specifically target females may reduce the risk of sex disparities in CVD care in Australia.

Machine learning models may also improve the accuracy of risk identification for individuals with Type 2 diabetes, a group with an elevated risk of CVD [29], compared to the non-diabetic population. The 10% increase in the sensitivity of the risk assessment for subgroups with diabetes found in this analysis suggests an opportunity to optimize and individualize cardiovascular risk reduction interventions for individuals with diabetes. 

A Synthetic Minority Oversampling Technique (SMOTE) was used to address the class imbalance. The sample used in the analysis was sufficiently powered for machine learning modelling approaches, and SMOTE is an accepted method for treating imbalanced data [22]. 

With the growing number of electronic health record datasets, there is an opportunity to use machine learning techniques to improve the accuracy of models by enabling a more nuanced account of the complex relationships between multiple, correlated, and nonlinear risk factors and outcomes [10] and supporting an adaptive approach for risk predictor revisions [30]. Incorporated into decision making tools in general practice, machine learning models of CVD risk may offer more accurate information to guide clinicians’ recommendations for treatment for high risk individuals. Intensive risk factor management can potentially lead to a reduction in CVD events and, particularly, of nonfatal myocardial infarction, stroke, and CVD death [2]. 

This analysis combined data collected across the studies of three prospective cohorts. One limitation of this approach is that it is possible there are unknown inaccuracies in this data, in the recorded cause of death, and self-reported variables (smoking status, diabetes, and use of medication). There is also known missing data in these datasets (32% of HDL cholesterol data from the MCCS dataset was missing). Missing HDL cholesterol data was imputed using a random forest-based imputation method, which can perform well with even a high amount of missing data [31], but imputing large proportions of missing data runs the risk of potentially biasing the model. Additionally, although the study cohorts are broadly representative of the wider Australian population, in all cohorts, non-English speakers who did not have access to support from an English language speaker were excluded from the studies and the MCCS participants were more likely to be older, female, and European-born than other Australians of the same age range [11]. 

For the purposes of comparison, the analysis approach utilized in this investigation included only the eight key health parameters identified in the Framingham model, developed in 2008, as these factors are routinely included in databases. This may limit the predictive accuracy of our models. Recently established CVD predictors, particularly those associated with elevated CVD risk in females or individuals with history of diabetes, should be included in future databases and investigations of machine learning models. In addition, the Framingham risk model is used to assess cardiovascular disease risk, while in this study we assessed only CVD mortality risk, not CVD incidence because CVD incidence information was not available in all of the three included datasets. Lastly, we did not recalibrate the Framingham model to the Australian dataset as we wanted to compare the machine learning model with the exact same model as recommended by the Framingham 2008 equation [21].

## 5. Conclusions

In this study, we developed machine learning risk prediction models for CVD mortality based on data from three popular Australian cohort studies using the same eight risk variables used by the Framingham 2008 model. The machine learning risk prediction models were significantly better than the traditional Framingham risk model for predicting CVD mortality risk in the Australian population. Machine learning models outperformed Framingham in each of the individual study cohorts, and in the combined cohort. Machine learning models also outperformed Framingham when stratified by sex and by diabetic diagnosis. Our findings suggest that machine learning models should be considered in the development of standard CVD risk assessment scales in future.

## Figures and Tables

**Table 1:** Data collection methods and measures for the cardiovascular disease (CVD) risk factor variables used in the analysis.

Risk Factor	Data Collection Methods	Measures
Age	Self-report	Years
Sex	Self-report	Male/Female
Total Cholesterol	Biomedical measure	Fasting blood sample Lipids
High-density lipoprotein (HDL) Cholesterol		
Systolic blood pressure	Biomedical measure	Dinamap/mercury sphygmomanometer, average of two recorded measures
Hypertension medication	Self-report	No/Yes
Diabetes	Self-report or biological measure	Told by a doctor that they have diabetesFasting plasma glucose (FPG) level of at least 7.0 mmol/L
Smoking status	Self-report	No/Yes

**Table 2:** Missing numbers and summary data (mean ± standard deviation) for the three-study cohorts and combined cohort. The values for n, age, male, female, total cholesterol, HDL cholesterol, systolic blood pressure, hypertension medication, diabetes, and smoker were input after removing CVD history and death, missing data, and imputation of other missing risk factor variables.

	North West Adelaide Health Study (NWAHS)		Australian Diabetes, Obesity, and Lifestyle (AusDiab)		Melbourne Collaborative Cohort Study (MCCS)		Combined	
	Summary	Missing	Summary	Missing	Summary	Missing	Summary	Missing
n	3654		10,150		32,611		46,305	
Age, y	48.5 ± 15.8	nil	50.0 ± 7.5	nil	54.4 ± 8.6	nil	53.0 ± 10.9	nil
Male, n%	1693 (46.3)	nil	4437 (43.7)	nil	12,790 (39.3)	nil	18,919 (40.8)	nil
Female, n%	1961 (53.7)	nil	5713 (56.3)	nil	19,722 (60.7)	nil	27,386 (59.2)	nil
Total cholesterol (mg/dL)	94.9 ± 18.8	41	102.1 ± 23.4	2	99.2 ± 19.0	151	99.5 ± 19.1	194
HDL cholesterol (mg/dL)	24.7 ± 6.8	41	25.8 ± 1.6	4	29.4 ± 7.9	10,503	29.7 ± 42.4	10,548
Systolic blood pressure (mm Hg)	126.6 ± 17.9	0	128.4 ± 7.5	54	135.9 ± 18.7	117	133.5 ± 18.9	171
Hypertension medication, n%	451 (12.3)	0	792 (7.8)	98	4671 (14.4)	94	6452(13.9)	192
Diabetes, n%	233 (6.4)	13	1252 (12.3)	169	1051 (3.2)	9	3791(8.2)	191
Smoker	1957 (53.6)	22	2124 (20.9)	212	13,382 (41.2)	10	19,833(42.8)	244
History of CVD	326	6	938	142	7035	nil	8299	148
CVD death, n%	121 (3.3)	70	341 (3.4)	17	520 (1.6)	1867	982(2.1)	1954

**Table 3:** Two-fold cross validation: Comparison of the performance of Framingham Score (baseline model (BL)) and four machine learning (ML) models predicting 15-year risk of CVD mortality of NWAHS, AusDiab and MCCS participants, and combined cohorts.

Models	Area-under-curve (AUC) (95% CI)	*p* Value	Difference from Framingham
NWAHS			
BL: Framingham Score	0.837 (0.792–0.882)	–	–
ML: Logistic Regression	0.874 (0.833–0.915)	<0.001	+3.7%
ML: Linear Discriminant Analysis	0.874 (0.833–0.915)	<0.001	+3.7%
ML: Support Vector Machine	0.873 (0.832–0.914)	<0.001	+3.6%
ML: Random Forest	0.854 (0.811–0.897)	0.0162	+1.7%
AusDiab			
BL: Framingham Score	0.850 (0.824–0.876)	–	–
ML: Logistic Regression	0.900 (0.878–0.922)	<0.001	+5.0%
ML: Linear Discriminant Analysis	0.901 (0.879–0.923)	<0.001	+5.1%
ML: Support Vector Machine	0.902 (0.880–0.924)	<0.001	+5.2%
ML: Random Forest	0.891 (0.868–0.914)	<0.001	+4.1%
MCCS			
BL: Framingham Score	0.754 (0.730–0.778)	–	–
ML: Logistic Regression	0.753 (0.729–0.777)	0.230	−0.1%
ML: Linear Discriminant Analysis	0.756 (0.732–0.780)	0.070	+0.2%
ML: Support Vector Machine	0.758 (0.734–0.782)	0.008	+0.4%
ML: Random Forest	0.781 (0.757–0.805)	<0.001	+2.7%
Combined			
BL: Framingham Score	0.802 (0.783–0.817)		–
ML: Logistic Regression	0.852 (0.837–0.867)	<0.001	+5.1%
ML: Linear Discriminant Analysis	0.852 (0.837–0.867)	<0.001	+5.1%
ML: Support Vector Machine	0.851 (0.836–0.866)	<0.001	+5.1%
ML: Random Forest	0.832 (0.814–0.848)	0.001	+3.0%

**Table 4:** Two-fold cross validation: Comparison of classification (Sensitivity, Specificity, Precision) and net reclassification improvement (NRI) performance of Framingham Score (baseline model (BL)) and four machine learning (ML) models predicting 15-year risk of CVD mortality of NWAHS, AusDiab and MCCS participants, and the combined cohort.

Models	Sensitivity	Specificity	Precision	NRI % (95%)	*p* Value
NWAHS					
BL: Framingham Score	41.3	91.3	14.0	–	
ML: Logistic Regression	79.5	81.7	13.2	28.5 (25.9–30.5)	<0.001
ML: Linear Discriminant Analysis	77.7	84.1	14.5	29.1 (26.1–30.6)	<0.001
ML: Support Vector Machine	80.7	81.0	12.9	29.0 (26.0–31.8)	<0.001
ML: Random Forest	79.4	80.8	12.7	27.5 (25.7–29.6)	<0.001
AusDiab					
BL: Framingham Score	57.1	88.2	14.4	–	
ML: Logistic Regression	84.6	84.1	16.1	23.3 (21.1–25.2)	<0.001
ML: Linear Discriminant Analysis	85.2	84.0	15.7	23.8 (20.7–26.1)	<0.001
ML: Support Vector Machine	84.0	85.4	16.7	24.1 (22.7–27.7)	<0.001
ML: Random Forest	84.3	83.6	15.3	22.5 (20.5–24.4)	<0.001
MCCS					
BL: Framingham Score	31.2	91.4	5.6	–	
ML: Logistic Regression	71.1	68.4	3.5	16.9 (13.6–19.9)	<0.001
ML: Linear Discriminant Analysis	70.4	69.5	3.6	17.3 (14.1–20.2)	<0.001
ML: Support Vector Machine	72.0	68.1	3.6	17.5 (13.6–20.4)	<0.001
ML: Random Forest	81.6	63.1	3.5	22.1 (19.1–24.8)	<0.001
Combined					
BL: Framingham Score	41.5	90.7	8.8	–	
ML: Logistic Regression	81.0	77.7	8.1	26.5 (20.1–29.8)	<0.001
ML: Linear Discriminant Analysis	80.5	78.2	8.2	26.5 (20.0–29.9)	<0.001
ML: Support Vector Machine	80.8	77.8	8.1	26.4 (19.8–29.5)	<0.001
ML: Random Forest	77.4	76.9	6.8	22.0 (16.5–27.5)	<0.001

**Table 5:** Variable ranking based on their contribution to the prediction for NWAHS, AusDiab, and MCCS populations. Variables are listed based on their contribution (Score) to the predictions.

NWAHS		AusDiab		MCCS		Combined	
*Variable*	*Score*	*Variable*	*Score*	*Variable*	*Score*	*Variable*	*Score*
Age	0.412	Age	0.429	Age	0.422	Age	0.563
Systolic blood pressure	0.251	Systolic blood pressure	0.301	Systolic blood pressure	0.222	Systolic blood pressure	0.201
Hypertension Medication	0.141	Hypertension medication	0.116	Hypertension medication	0.141	Hypertension medication	0.125
Diabetes status	0.089	Diabetes status	0.077	HDL	0.105	Diabetes status	0.070
Tot. Cholesterol	0.057	HDL	0.036	Tot. Cholesterol	0.066	HDL	0.020
HDL	0.028	Tot. Cholesterol	0.028	Diabetes status	0.032	Sex	0.011
Sex	0.011	Sex	0.008	Sex	0.005	Tot. Cholesterol	0.008
Smoking status	0.010	Smoking status	0.004	Smoking status	0.004	Smoking status	0.005

**Table 6:** Two-fold cross validation: Comparison of the performance of Framingham Score (baseline model (BL)) and four machine learning (ML) models predicting 15-year risk of CVD mortality using combined data based on Sex stratification.

Models	AUC (95% CI)	*p* Value	Difference from Framingham
Men			
BL: Framingham Score	0.799 (0.776–0.823)	–	–
ML: Logistic Regression	0.816 (0.793–0.839)	<0.001	+1.7%
ML: Linear Discriminant Analysis	0.818 (0.795–0.841)	<0.001	+1.9%
ML: Support Vector Machine	0.818 (0.795–0.841)	<0.001	+1.9%
ML: Random Forest	0.812(0.791–0.837)	<0.001	+1.7%
Women			
BL: Framingham Score	0.836 (0.814–0.858)	–	–
ML: Logistic Regression	0.871 (0.851–0.892)	<0.001	+3.5%
ML: Linear Discriminant Analysis	0.869 (0.848–0.890)	<0.001	+3.4%
ML: Support Vector Machine	0.870 (0.850–0.891)	<0.001	+3.4%
ML: Random Forest	0.854 (0.833–0.876)	< 0.001	+2.0%

**Table 7:** Two-fold cross validation: Comparison of the classification (Sensitivity, Specificity, Precision) and NRI performance of Framingham Score (baseline model (BL)) and four machine learning (ML) models predicting 15-year risk of CVD mortality using combined data based on Sex stratification.

Models	Sensitivity	Specificity	Precision	NRI % (95%)	*p* Value
Men					
BL: Framingham Score	66.3	79.3	8.0	–	
ML: Logistic Regression	75.9	75.8	8.6	6.1 (5.0–8.4)	<0.001
ML: Linear Discriminant Analysis	76.2	75.5	8.8	6.1 (5.0–8.8)	<0.001
ML: Support Vector Machine	76.1	76.0	8.6	6.5 (6.1–7.7)	<0.001
ML: Random Forest	77.1	74.0	7.6	5.5 (4.0–6.4)	<0.001
Women					
BL: Framingham Score	15.6	98.5	15.7	–	
ML: Logistic Regression	83.4	79.1	7.7	48.4 (46.4–50.1)	<0.001
ML: Linear Discriminant Analysis	81.9	80.8	8.6	48.7 (46.0–50.0)	<0.001
ML: Support Vector Machine	83.4	79.4	8.1	48.7 (47.3–49.6)	<0.001
ML: Random Forest	80.6	77.6	6.1	44.1 (43.6–46.5)	<0.001

**Table 8:** Two-fold cross validation: Comparison of the performance of Framingham Score (baseline model (BL)) and four machine learning (ML) models predicting 15-year risk of CVD mortality using combined data based on diabetes stratification.

Models	AUC (95% CI)	*p* Value	Difference from Framingham
Diabetes			
BL: Framingham Score	0.734 (0.696–0.771)	–	–
ML: Logistic Regression	0.823 (0.790–0.856)	<0.001	+9.0%
ML: Linear Discriminant Analysis	0.824 (0.791–0.857)	<0.001	+9.1%
ML: Support Vector Machine	0.824 (0.791–0.857)	<0.001	+9.0%
ML: Random Forest	0.800 (0.766–0.835)	<0.001	+6.6%
Non-Diabetes			
BL: Framingham Score	0.789 (0.770–0.88)	–	–
ML: Logistic Regression	0.842 (0.824–0.860)	<0.001	+5.3%
ML: Linear Discriminant Analysis	0.843 (0.825–0.861)	<0.001	+5.4%
ML: Support Vector Machine	0.844 (0.826–0.862)	<0.001	+5.5%
ML: Random Forest	0.831 (0.813–0.850)	<0.001	+4.2%

**Table 9:** Two-fold cross validation: Comparison of the classification (Sensitivity, Specificity, Precision) and NRI performance of Framingham Score (baseline model (BL)) and four machine learning (ML) models predicting 15-year risk of CVD mortality using combined data based on diabetes stratification.

Models	Sensitivity	Specificity	Precision	NRI % (95%)	*p* Value
Diabetes					
BL: Framingham Score	70.1	63.4	11.1	–	
ML: Logistic Regression	78.8	72.7	16.0	17.9 (15.1–19.6)	<0.001
ML: Linear Discriminant Analysis	80.0	72.2	16.0	18.7 (16.9–20.0)	<0.001
ML: Support Vector Machine	79.6	72.2	15.8	18.2 (15.6–20.0)	<0.001
ML: Random Forest	79.7	70.7	15.3	16.8 (14.5–19.2)	<0.001
Non-Diabetes					
BL: Framingham Score	32.6	93.0	7.7	–	
ML: Logistic Regression	81.2	75.3	5.7	30.8 (28.6–34.2)	<0.001
ML: Linear Discriminant Analysis	83.7	73.1	5.6	31.2 (27.6–34.4)	<0.001
ML: Support Vector Machine	80.2	76.2	6.6	30.8 (28.7–34.0)	<0.001
ML: Random Forest	77.4	76.7	5.7	28.5 (26.4–32.5)	<0.001

**Table 10:** External Validation: Comparison of the classification and NRI performance of Framingham Score (baseline model (BL)) and four machine learning (ML) models predicting 15-year risk of CVD mortality using combined AusDiab and MCCS dataset as the training set and NWAHS as the external validation set.

Models	AUC	Sensitivity	Specificity	Precision	NRI

BL: Framingham Score	0.837	41.3	91.3	14.0	-
ML: Logistic Regression	0.879	76.0	85.7	15.4	29.1
ML: Linear Discriminant Analysis	0.880	75.2	86.8	16.4	29.4
ML: Support Vector Machine	0.880	72.5	89.0	18.5	28.9
ML: Random Forest	0.866	79.4	80.4	12.2	27.2
Men					
BL: Framingham Score	0.841	72.1	82.4	13.3	-
ML: Logistic Regression	0.858	73.8	83.8	14.6	3.1
ML: Linear Discriminant Analysis	0.857	73.7	83.5	14.3	2.7
ML: Support Vector Machine	0.856	73.9	84.6	14.8	1.3
ML: Random Forest	0.846	72.13	82.65	13.5	0.28
Women					
BL: Framingham Score	0.871	10.5	97.4	22.2	-
ML: Logistic Regression	0.898	87.3	78.8	11.6	58.2
ML: Linear Discriminant Analysis	0.898	88.1	78.6	11.7	58.8
ML: Support Vector Machine	0.900	88.4	78.4	13.5	58.9
ML: Random Forest	0.891	84.5	83.1	11.6	59.7
Diabetes					
BL: Framingham Score	0.675	66.7	57.8	15.3	-
ML: Logistic Regression	0.744	74.4	71.4	23.1	21.3
ML: Linear Discriminant Analysis	0.741	75.0	70.5	22.5	21.0
ML: Support Vector Machine	0.738	75.8	65.3	19.8	16.6
ML: Random Forest	0.706	62.5	79.1	25.4	17.1
Non-Diabetes					
BL: Framingham Score	0.841	35.1	93.4	13.5	-
ML: Logistic Regression	0.889	80.4	83.6	12.5	35.5
ML: Linear Discriminant Analysis	0.888	83.5	80.4	11.1	35.4
ML: Support Vector Machine	0.890	87.6	76.0	9.7	35.1
ML: Random Forest	0.866	78.4	81.9	11.0	31.8

## Data Availability

Restrictions apply to the availability of these data. Data was obtained from the North West Adelaide Health Study; the Australian Diabetes, Obesity, and Life-style study; and the Melbourne Collaborative Cohort Study.
